# Soil nutrients and precipitation are major drivers of global patterns of grass leaf silicification

**DOI:** 10.1002/ecy.3006

**Published:** 2020-04-17

**Authors:** Kathleen M. Quigley, Daniel M. Griffith, George L. Donati, T. Michael Anderson

**Affiliations:** ^1^ Department of Biology Wake Forest University Winston‐Salem North Carolina 27109 USA; ^2^ Department of Chemistry Wake Forest University Winston‐Salem North Carolina 27109 USA; ^3^ Present address: Department of Plant, Soil and Microbial Sciences Michigan State University Plant, Soil, and Microbial Sciences Building, 1066 Bogue Street, Room A286 East Lansing Michigan 48824 USA; ^4^ Present address: Department of Forest Ecosystems and Society Oregon State University Corvallis, Oregon 97333 USA

**Keywords:** herbivory, leaf economics, phytolith, plant nutrition, resource availability, silica, soil nutrients, stoichiometry

## Abstract

Grasses accumulate high concentrations of silicon (Si) in their tissues, with potential benefits including herbivore defense, improved water balance, and reduced leaf construction costs. Although Si is one of the most widely varying leaf constituents among individuals, species, and ecosystems, the environmental forces driving this variation remain elusive and understudied. To understand relationships between environmental factors and grass Si accumulation better, we analyzed foliar chemistry of grasses from 17 globally distributed sites where nutrient inputs and grazing were manipulated. These sites span natural gradients in temperature, precipitation, and underlying soil properties, which allowed us to assess the relative importance of soil moisture and nutrients across variation in climate. Foliar Si concentration did not respond to large mammalian grazer exclusion, but significant variation in herbivore abundance among sites may have precluded the observation of defoliation effects at these sites. However, nutrient addition consistently reduced leaf Si, especially at sites with low soil nitrogen prior to nutrient addition. Additionally, a leaf‐level trade‐off between Si and carbon (C) existed that was stronger at arid sites than mesic sites. Our results suggest soil nutrient limitation favors investment in Si over C‐based leaf construction, and that fixing C is especially costly relative to assimilating Si when water is limiting. Our results demonstrate the importance of soil nutrients and precipitation as key drivers of global grass silicification patterns.

## Introduction

Silicon (Si) is unique among elements in that it accumulates in plant biomass to concentrations that exceed those of essential macronutrients, yet Si is considered nonessential (Epstein 1994). Plant roots absorb aqueous silicon (H_4_SiO_4_) from soil water, where its concentration often exceeds that of major inorganic nutrients like P, K, and Ca (Epstein 1994, Barber 1984). Si eventually polymerizes into solid silicon dioxide (SiO_2_ hereafter “silica”) within and between cells in aboveground plant tissue (Piperno 2006), a process that occurs concurrently with transpiration. However, the magnitude of silicification varies greatly among plant taxonomic groups. Si concentrations are near‐zero in legumes and most gymnosperms (Jones and Handreck 1969, Parry and Winslow 1977), intermediate in terrestrial angiosperms (typically 0.5–1% dry weight; Ma and Takahashi 2002, Trembath‐Reichert et al. 2015), and reach 5–10% of plant dry weight in some grasses and early diverging land plants (e.g., bryophytes and pteridophytes). Despite this extreme variation within and among species, the ecological and evolutionary drivers of phytogenic silica accumulation are unclear.

Silicon plays a role in alleviating plant abiotic stress, including salinity (Yin et al. 2015), drought (Hattori et al. 2005), metal toxicity (Cunha and Nascimento 2008, Vaculík et al. 2012, Che et al. 2016), and solar irradiation (Fang et al. 2011, Yao et al. 2011). Plants may actively regulate Si uptake in response to natural enemies, ranging from fungal pathogens (Rémus‐Borel et al. 2005) to insect (Massey et al. 2006) and mammalian herbivores (e.g., McNaughton and Tarrants 1983). Silica “phytoliths” may provide mechanical defense against herbivory when deposited in and around plant cell walls, as indicated by a reduction in gut absorptive surface area in animals fed a silica‐rich diet (Wieczorek et al. 2015). Studies that focus on the role of Si as an antiherbivore defense are among the most widely cited.

Elongated teeth observed in Miocene hypsodonts are hypothesized as an adaptation for grass diets rich in abrasive silica (Stebbins 1981), leading to a putative “arms race” in which grasses evolved mechanisms for induced Si uptake to deter herbivory (McNaughton and Tarrants 1983, McNaughton et al. 1985). The adaptive role of silica as a deterrent against herbivory remains a pervasive and compelling hypothesis. For example, domestic mammals prefer low silica grasses (Massey et al. 2009), some plants increase Si uptake following defoliation (Massey et al. 2007, Reynolds et al. 2012), and high‐silica diets result in reduced fitness for insect and mammalian herbivores (Massey and Hartley 2006, Jambunathan et al. 2009). Antiherbivore effects of Si may even cascade to reduce predator performance (Ryalls et al. 2017).

Fossil evidence, however, contradicts the hypothesis that grass Si accumulation evolved primarily as an adaptive response to increased grazing pressure by large‐bodied herbivores (Stromberg et al. 2016). Moreover, induction of plant silica by herbivory is not consistently observed in modern studies (Quigley and Anderson 2014). Many factors play a role in whether Si uptake is induced, including damage thresholds (Reynolds et al. 2012) and plant species identity (Kindomihou et al. 2006, Garbuzov et al. 2011, Soininen et al. 2013, Hartley 2015). An alternative hypothesis for grass Si accumulation is that heavily silicified leaves are a response to resource‐poor, open environments into which grasses expanded in the Miocene (e.g., Coughenour 1985, Edwards and Smith, 2010) and that the antiherbivore benefits of silica are secondary. Si concentration is negatively related to leaf longevity, which suggests a functional role for Si as an energetically cheap substitute for carbon in short‐lived leaves (Cooke and Leishman 2011). If defense is the primary function of leaf Si, ecological theories, such as the resource availability hypothesis (Coley et al. 1985, Endara and Coley 2011) and leaf economic spectrum (Wright et al. 2004, Shipley et al. 2006), predict a positive relationship between leaf silica and longevity. From a molecular standpoint, leaf silicification is costly because Si cannot bond with organic compounds, cannot be remobilized after it polymerizes, and has a high mass cost relative to carbon (Cooke and Leishman 2011). Thus, Si may relate to other leaf properties in a way that ultimately affects the rate of return on energetic investment, especially for plants in resource‐limited conditions (i.e., nutrient‐poor or arid conditions).

To date, ecological studies of plant Si accumulation have focused on how environmental factors influence taxonomic and landscape‐scale variation in leaf Si, but have yet to examine interactions among environmental factors along climate gradients or within a stoichiometric context. Thus, our goals were (1) to experimentally test the relative importance of mammalian grazers and soil nutrients as contemporary drivers of grass silicification, (2) to determine if grass silicification varies systematically with climate, and (3) to examine the leaf‐level stoichiometry of Si relative to other nutrients, particularly C, at the global scale. To investigate how soil nutrients, herbivory, and climate influence grass Si concentration, we analyzed grass leaf samples collected from 17 grass‐dominated Nutrient Network (NutNet) sites across five continents, where soil nutrient availability (i.e., NPK) and herbivory were experimentally manipulated (Borer et al. 2014a). We tested for main effects of the experimental treatments with the a priori expectations that (1) leaf Si would be higher in grazed plots, relative to ungrazed plots protected by fencing, if grass Si accumulation is in fact induced by herbivory (McNaughton and Tarrants 1983, McNaughton et al. 1985), and that (2) leaf Si would decrease in plots supplemented with NPK fertilizer if resource availability determines leaf investment in Si vs. C. We also tested whether a trade‐off exists between alternative leaf construction strategies, with the expectation that the high cost of water loss linked to photosynthesis at arid sites would favor the construction of Si‐ over C‐rich leaves, thus strengthening the Si–C trade‐off at arid compared to mesic sites.

## Materials and Methods

### Study sites and experimental design

**Figure 1:**
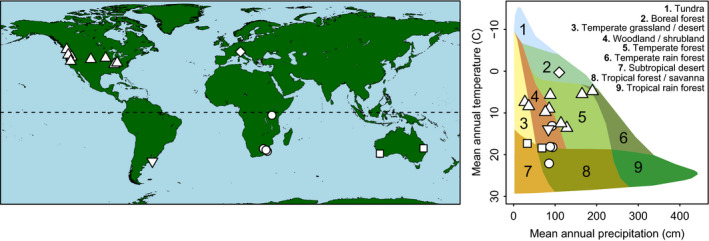
Global distribution and biome space of the 17 NutNet sites that were analyzed in this study. Overlapping points occur in North America (*n* = 8) and South Africa (*n* = 3). Although all sites are grasslands, they span far outside of the traditional (Whittaker) biome space (No. 4).

Data were collected from 17 sites within the Nutrient Network (NutNet hereafter), a coordinated research network with identical nutrient addition × grazer exclusion treatments executed since 2007 using a standard protocol (http://nutnet.umn.edu/). Sites represent a broad range of climate and biome space (mean annual precipitation [MAP]: 365–1,898 mm/yr; mean annual temperature [MAT]: 0.3–22.1°C) spanning five continents (Fig. 1; Appendix S1: Table S1). At each NutNet location (SITE), replicate blocks (2–5) each with 10 5 × 5 m plots were established within a footprint of >1,000 m^2^, and plots were randomly assigned to combinations of fertilization and grazer removal by fencing (O’Halloran 2013; see Appendix S1: Table S1 for site info). Treatments included a control (no fence, no nutrients added), nutrient‐addition plots (combinations of nitrogen, phosphorous, and potassium; NPK), fencing (FENCE), and FENCE + NPK. For our analysis, we focused on treatments representing the 2 × 2 factorial combination of grazing and full NPK fertilization: NPK, FENCE, FENCE + NPK, and the control plots. Nutrient treatments were applied yearly at 10 g m^−2^ yr^−1^ prior to the growing season, and an initial 100 g/m^2^ micronutrient mix (Fe, S, Mg, Mn, Cu, Zn, B, and Mo) was applied the first year only. Fences were constructed of heavy‐gauge wire to a height ≥1.2 m and secured at the base with wire mesh to 30 cm to exclude mammalian herbivores >50 g and digging, but not fully subterranean, animals (Borer et al. 2014b, 2019, Mortensen et al. 2018). A grazing index (range: 0–29; Appendix S1: Table S1), which served to weigh the relative intensity of grazing among sites, was calculated for each site by summing grazer importance values as reported by each site Principle investigator (PI) (Anderson et al. 2018). Soil samples were analyzed for texture, pH, soil C, and soil N at the plot level for most sites, as reported in Borer et al. 2014a. Aboveground standing biomass was collected ≥2 yr posttreatment from each plot by clipping to the ground and pooling two 10‐cm‐wide × 1‐m‐long strips of vegetation (BIOMASS). Samples were subdivided into six major vegetation types (litter, bryophytes, graminoids, legumes, forbs, and woody plants) on site, weighed, dried, and sent to Wake Forest University for analysis of leaf chemistry. Because sites were dominated by graminoids, and legumes and forbs had consistently very low Si concentrations, we focused subsequent analyses on graminoids.

### Measurement of leaf Si, C, and other elements

Leaf Si and C concentrations were estimated using near‐infrared spectroscopy (NIRS). Samples were ground using a cyclone mill (UDY Corp., Fort Collins, Colorado, USA), dried at 60°C for 48 h, then scanned in triplicate on a Bruker multipurpose NIRS (Bruker Optics Inc., Billerica, Massachusetts, USA). The Kennard–Stone algorithm (Kennard and Stone 1969), which identifies maximum variation among NIR spectra, was used to select ~ 20% of leaf samples (*n* = 345) for wet lab chemical analysis and subsequent entry into calibration models (Appendix S1: Methods S1). Si concentration of calibration samples was determined by inductively coupled plasma optical emission spectrometry (ICP OES) following autoclave digestion (Quigley et al. 2016), and leaf C was estimated by combustion on a CN 2000 combustion analyzer (LECO, Saint Joseph, Michigan, USA) at the Kansas State University soil testing laboratory. Standard qualitative metrics confirmed that the ICP OES reference method provided high within‐sample accuracy (SE of 12 replicate samples = 0.019%) and a low limit of detection (10 g/L). Leaf C, N, P, K, Mg, and Ca were estimated as described in detail by Anderson et al. (2018); model validation indicated an *R* ^2^ of ≥0.77 for all elements (range: 0.77 for P to 0.95 for N). A partial‐least‐squares (PLS) regression model fit to training data for silicon performed well in external validation (*R^2^* = 83%; Appendix S1: Methods S1, Fig. S1).

### Data analysis

Statistical analyses were conducted in version 3.4.2 of the R statistical programming environment (R Development Core Team 2018). The response variable of interest, leaf Si (% dry weight), exhibited a significant right skew (*A* = 4.13, *P* = 2.66e^−10^, from an Anderson‐Darling test) and was log‐transformed to meet assumptions of normality. To ensure homogeneity of variance and normality of error, we used the “ncvTest” function in the *cars* package (Fox and Weisberg 2019.) and graphically assessed histograms of residuals.

We constructed linear mixed‐effects models with grass leaf Si as the response variable, SITE as a random effect, and experimental treatments (NPK and FENCE), climate variables (mean annual precipitation [MAP], mean annual temperature [MAT], and potential evapotranspiration [PET]), and soil conditions (particle size distribution [SAND], pH, organic matter [OM], total carbon [soil C] and total nitrogen [soil N]) as fixed effects. See Anderson et al. (2018) for a full description of how climate and soil variables were derived for each site. Models were fit using the R package *lme4* (Bates et al. 2015), and the conditional and marginal coefficients of determination were calculated using the R package *MuMIn* (Nakagawa and Schielzeth 2013, Barton 2019).

We began with a two‐way mixed‐model analysis with SITE as a random effect and FENCE, NPK and their interaction, as main effects (*n* = 15 sites with both fence and fertilizer treatments). We used the “lsmeansLT” command in the R package *lmerTest* to calculate mean responses after accounting for random effects (Kuznetsova et al. 2017). Subsequently, we used a multimodel inference approach, based on Akaike’s information criterion (AIC), to identify the top model(s) predicting grass leaf Si from experimental treatments and site‐level covariates (*n* = 17 sites). Included in our model selection were all additive models and, where logical, two‐way interactions among predictors. Models with a ΔAIC < 2 were considered equivalent (Burnham and Anderson 2004), and ΔAIC values above 7 indicated poor fit. For relationships between leaf Si and environmental predictors which appeared to be nonlinear, such as between leaf Si and soil N, we used the R package *rpart* to perform regression tree analysis (Thernau and Atkinson 2019). This method groups observations based on explanatory variables to identify the predictors that explain variation in nonlinear response variables (De’Ath and Fabricius 2000). To avoid over‐fitting regression trees, we used a “one‐standard error” rule in which the most parsimonious model within one standard error of the best model was selected (Hastie, Tibshirani and Friedman 2009).

Finally, we tested the hypothesis that Si and C trade off in response to resource availability by comparing the Si ~ C relationship to the relationship between Si and other elements. We considered the alternative hypothesis that variation in leaf %Si is constrained to decrease as %C increases because element concentrations must sum to 1 (an effect we call the “stoichiometric dilution effect”), rather than from a trade‐off whereby Si explicitly replaces C. This is a key distinction, because increases in %C inherently result in decreases in the concentrations of all other elements. Consequently, we compared the observed slope of the %Si ~ %C relationship to a null expectation based on a simulated stoichiometric dilution effect and then repeated this for the additional macronutrients (N, P, K, Ca, and Mg). For a given focal element (e.g., Si), each %C value (*N* = 457) was paired with a random concentration from a nonfocal element of the same sample (i.e., for focal element Si, either N, P, K, Ca, or Mg was selected at random). The slope from a linear mixed effects model (with SITE as a random effect) that related the random element to %C was then estimated, and the process was repeated 999 times to produce a distribution of random slopes for each focal element. The observed slope between %C and a focal element was then compared to the distribution of random slopes, which represents the mean effect of C on other elements. A focal element slope more negative than expected suggests that an element may trade off with C, whereas a focal element slope greater than or equal to the predicted slope suggests that the element concentration varies as predicted by the stoichiometric dilution effect.

## Results

### Drivers of leaf silica across sites

**Figure 2:**
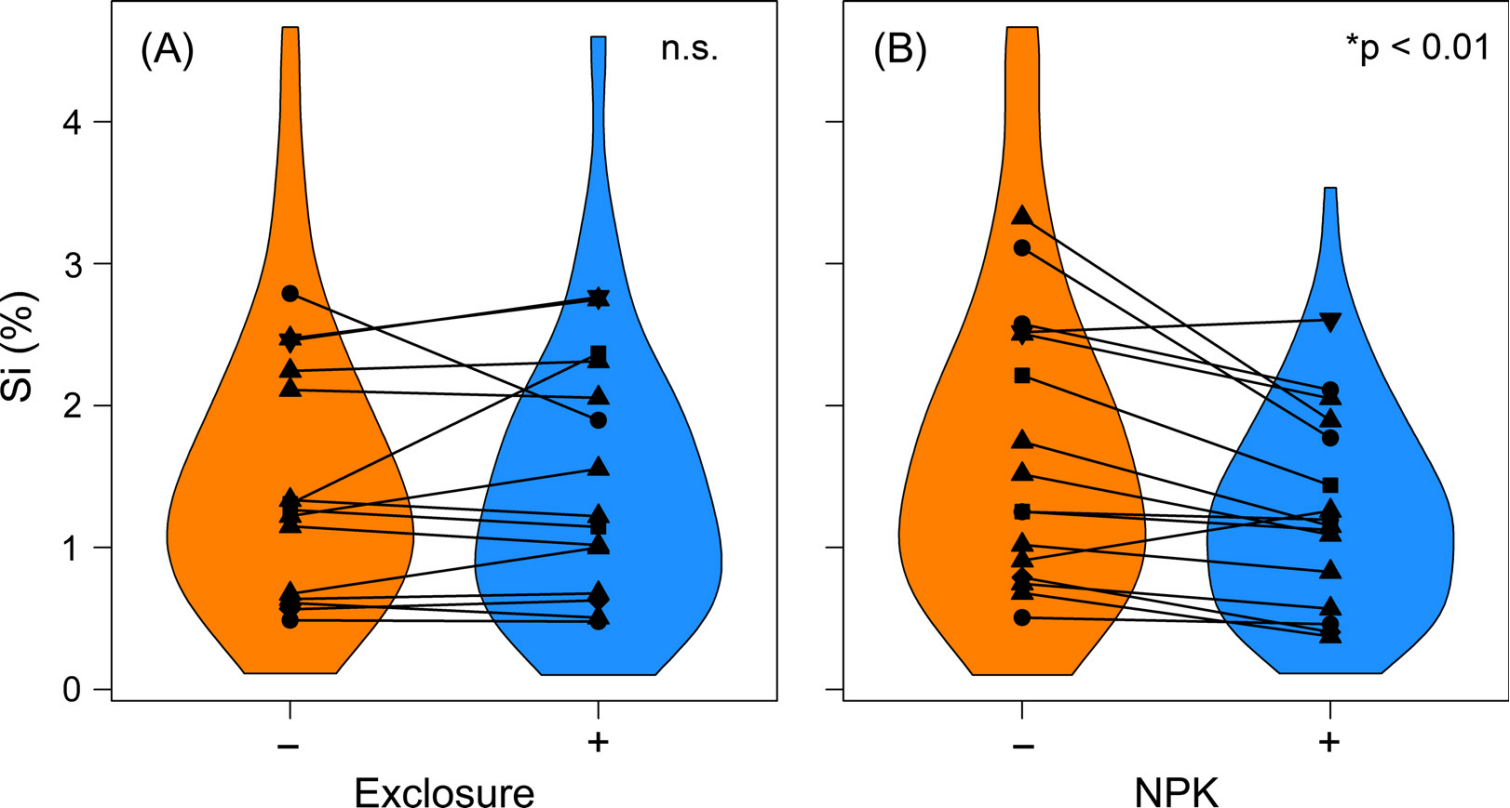
Leaf Si did not differ significantly inside and outside of grazing exclosures (A), but a significant decline in leaf Si was observed following the addition of NPK fertilizer (B). Violin plots show the distribution of all leaf Si values; points represent mean site values across blocks; dashed lines represent mean leaf Si (%) at sites. Panel (A) excludes two sites (Summerveld and Ukulinga; *n* = 15) that did not have exclosures constructed, and panel (B) excludes one site (Hall; *n* = 16) that did not have grass samples representing full +/− NPK treatments.

In our two‐way analysis, experimental fertilization had a strong and consistent negative effect on plant Si (estimate = −0.49 ± 0.11, *t* = −4.32, *P* < 0.001). Plant Si decreased in response to NPK addition at 12 out of 15 sites, did not respond at 1, and increased at 2 sites (Fig. 2). After accounting for random intercepts of sites, leaf Si concentration was significantly greater in control plots than NPK addition plots (−NPK mean [Si] ± SD = 1.72 ± 0.19% dry weight (dw), +NPK mean [Si] ± SD = 1.27 ± 0.19% dw). In contrast, experimental manipulation of grazers by fencing had weak and inconsistent effects on plant Si (estimate = −0.015 ± 0.13, *t* = −0.12, *P* = 0.91). In response to fencing, plant Si increased at five sites, decreased at four, and had a negligible response at the remaining sites (Fig. 2). In addition, there was no evidence for a fencing by fertilization interaction in the two‐way analysis (estimate = 0.08 ± 0.17, *t* = 0.47, *P* = 0.64).

**Figure 3:**
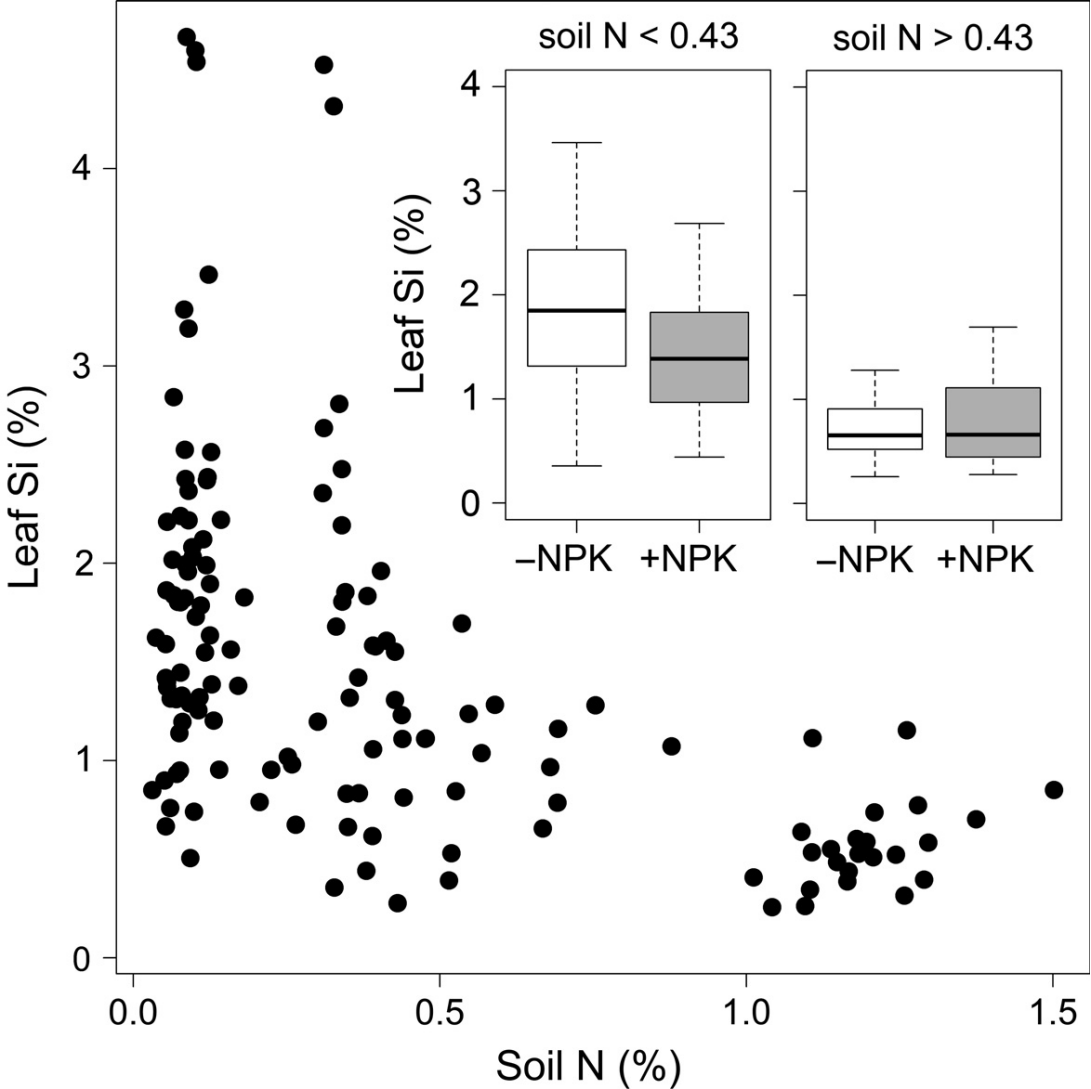
Grass leaf silicon plotted against total soil nitrogen. Inset boxplots illustrate that the NPK response occurred above a threshold of soil N (N < 0.43%). A mixed model that included soil N in addition to NPK treatment provided the greatest predictive strength (Akaike’s information criterion score) for leaf silicon (Table 1).

In our comprehensive model selection analysis, soil nitrogen and NPK treatment were the two best predictors of grass leaf silicon. Regression tree analysis indicated that NPK addition was a particularly important predictor of leaf Si concentration below a soil nitrogen threshold of 0.43% N (Fig. 3 inset; complexity parameter = 0.034). Leaf Si showed the greatest variation at sites with low soil nitrogen, and very little variation in leaf Si was observed above the soil N threshold (0.43%; Fig. 3). After accounting for differences among sites, NPK treatment explained ~5% of the variation in leaf Si (ΔAIC = 2.5), whereas the optimal model including NPK + soil N explained 23% of variation in leaf Si (Appendix S1: Table S2). Like the two‐way analysis, the fencing treatment was not included in any model with a ΔAIC < 7, nor was the grazing index part of any of the competitive models. No alternative models were equivalent to NPK + soil N, which had a 60.4% probability of being the best of the model set (Table 1, *w_i_*). Furthermore, models including NPK as an explanatory variable had consistently low ΔAIC values, and models including herbivory (fence or grazing index) were consistently poor predictors of leaf Si (Appendix S1: Table S2). One possible explanation for the lack of a clear fencing effect (discussed below) is the significant variation in large herbivore biomass and rates of consumption across the NutNet sites.

**Table 1:** Summary of models within 5 ΔAIC of the best‐fit model for predicting grass leaf Si. Predictor variables in the full model comparison (Appendix S1: Table S2) included: nutrient addition (NPK), grazer removal (fence), soil N, soil C, soil pH, grazing index, soil % sand, mean annual precipitation (MAP), and mean annual temperature (MAT). The response variable (Si) was log transformed in all models to correct for its skewed distribution. Models are ranked according to Akaike’s information criterion (AIC) score. *R* ^2^_m_ represents the coefficient of determination for fixed effects only (marginal), and *R* ^2^_c_ represents the coefficient of determination which accounts for a random effect of site (conditional), as described by Nakagawa and Schielzeth (2013). The Akaike weight (*w_i_*) indicates the probability that a model from the respective set is the best one. See Appendix S1: Table S2 for full model comparison list.

Model components	df	AIC	ΔAIC	*w_i_*	*R* ^2^_m_	*R* ^2^_c_
NPK + soil N	5	166.02	0.0	0.60	0.23	0.66
NPK	4	168.64	2.5	0.16	0.05	0.71
NPK + soil C	5	169.60	3.6	0.10	0.25	0.68
NPK * soil N	6	169.60	3.7	0.10	0.22	0.66

### Foliar stoichiometry

**Figure 4:**
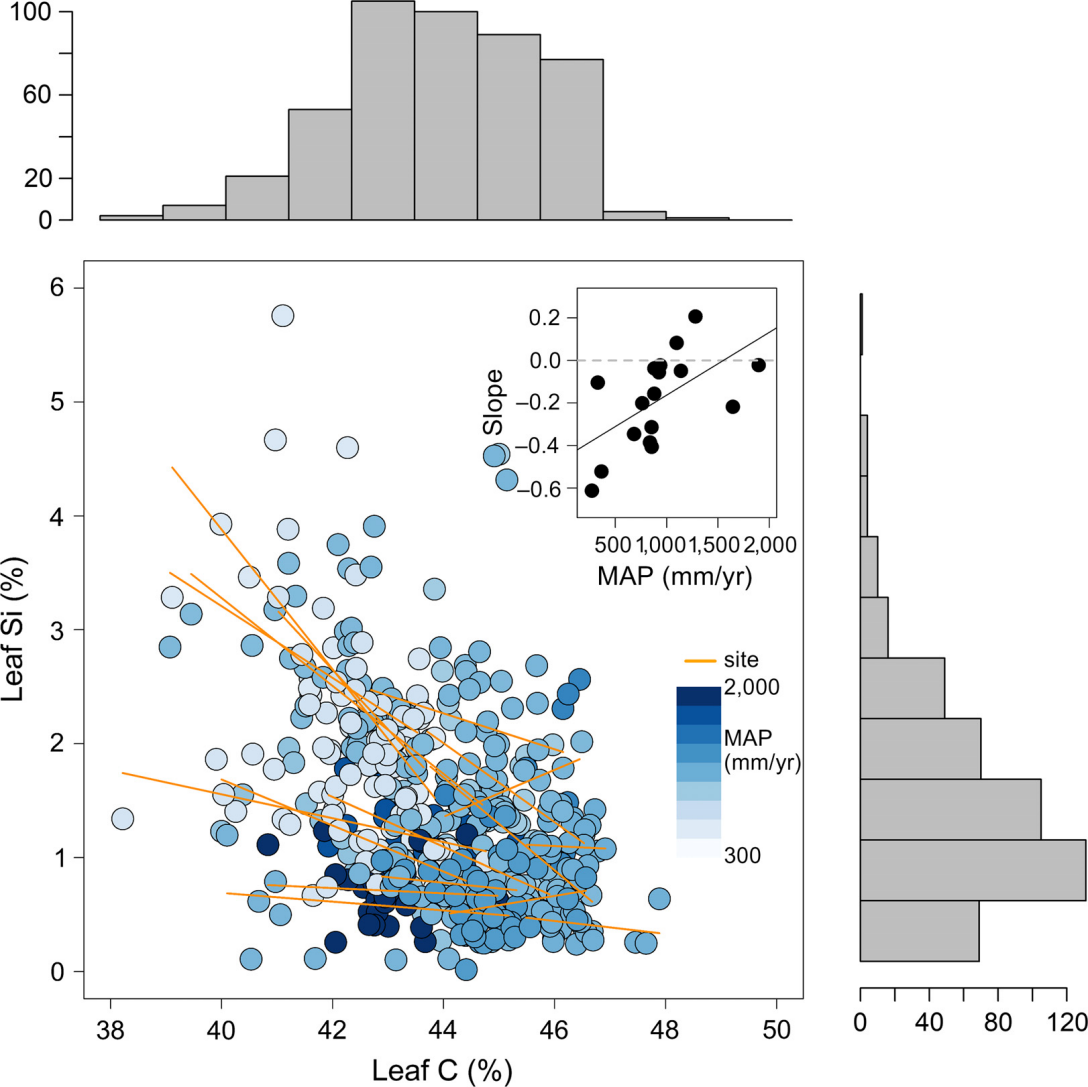
Leaf silicon exhibited a strong negative correlation with leaf carbon, especially at arid sites. Each point represents an individual grass sample; points are colored according to site‐specific precipitation (darker blue indicates greater precipitation). Orange lines represent site‐specific regressions between leaf Si and leaf C. Inset: each point represents the slope between leaf Si and leaf C in relation to precipitation at one site. The strength of the negative relationship between Si and C weakened as site precipitation increased. Gray dashed line represents the null hypothesis of no correlation between precipitation and the leaf Si ~ leaf C trade‐off.

Across sites, grass leaf carbon varied from 38 to 48% and demonstrated a negative relationship with grass leaf Si concentration (Fig. 4). We tested the strength of leaf Si ~ leaf C coefficient against environmental variables, including PET, MAT, and MAP, which revealed that the sign and strength of within‐site Si ~ C slopes sites varied systematically with MAP. At arid sites, the linear correlation between Si and C was strongly negative, whereas the slopes of correlations weakened along a gradient of increasing precipitation (Fig. 4 inset). Results of our model simulation indicated that the strength of the negative slope between leaf C and leaf Si was greater than would be expected by the stoichiometric dilution effect (Appendix S1: Fig. S2, panel A). In contrast, the slopes for the remaining elements (N, P, K, Ca, and Mg) were equal to, or less than, predicted by the model simulation (Appendix S1: Fig. S2, panels B–F), suggesting that these macronutrients all behaved as predicted by a stochiometric dilution effect.

## Discussion

Across 17 globally distributed grassland sites, nutrient addition and soil nutrient status elicited the strongest response in grass leaf Si. N fertilization has been linked to decreased grass leaf Si (Jones and Handreck 1967, Street 1974, Wallace et al. 1976, Gali‐Muhtasib et al. 1992), but little consideration has been given to the processes driving this pattern. We considered three possible mechanistic explanations. First, stoichiometric dilution may result in decreased leaf Si, as has been observed for leaf N, P, and K concentrations following NPK addition (Anderson et al. 2018). However, all macronutrients tested via our model simulation (N, P, K, Ca, and Mg) varied with leaf C in a manner as predicted by stoichiometric dilution, except for Si. This suggests that although mass balance for essential elements is tightly regulated above a minimum requirement, nonessential elements, such as Si, vary more widely. Second, changes in species composition may have caused decreased plot‐level Si. We explored this option at one site (Chichaqua Bottoms) where grasses were identified to species (Appendix S1: Table S1). Linear mixed models indicated that species had no main effect on leaf Si accumulation after accounting for NPK. However, including species as a random effect did improve model fit, suggesting that grass species differ in baseline Si, but their leaf Si responds similarly to NPK addition. For example, *Andropogon gerardii* (C4) at Chichaqua Bottoms had significantly lower Si concentration than all C_3_ species measured (Appendix S1: Fig. S3). Although anecdotal, this suggests that C_4_ species have a decreased demand for Si investment because of greater C assimilation efficiency. Third, NPK addition may improve plant carbon use efficiency leading to decreased Si. Under nutrient‐poor conditions, Si may act as a metabolically cheap C replacement, allowing optimal C allocation toward growth while maintaining structural attributes conferred by silica. A negative relationship between leaf Si and leaf life span implies a role for Si in the leaf economics spectrum as a C replacement in short‐lived leaves (Cooke and Leishman 2011). Although relative investment in herbivore defense is also linked to leaf life span (Coley et al. 1985), the defensive role of Si in the resource availability hypothesis remains uncertain. Regardless of the biological role of leaf Si, soil resource availability alters grass allocation in C vs. Si.

Negative correlations have been reported between leaf silica and carbon‐based compounds (lignin, cellulose, phenols) and, more broadly, between silica and total leaf C (Schoelynck et al. 2010, Cooke and Leishman 2012, Neu et al. 2017, Klotzbücher et al. 2018). One study suggested that silicon was not acting as a structural substitute for C, because, although a negative correlation was observed between leaf Si and total C, no relationship was observed between Si and lignin or cellulose (Cooke and Leishman 2012). The same study also reported that high Si plants had decreased phenolic and tannin concentrations, suggesting a trade‐off in the type of defense used, rather than between growth and defense per se. However, the results were for 11 plant families (47 species), most of which do not exhibit the extreme within‐taxon leaf Si variation observed in Poaceae and therefore do not reflect the full costs of Si accumulation. Although silica is metabolically inexpensive relative to lignin (Raven 1983), it cannot be remobilized and transported in phloem like other plant nutrients (Loneragan et al. 1976). The rigid and immobile nature of phytoliths is assumed to incur a cost (Cooke and Leishman 2012), but investigations of physiological plant traits, such as leaf or stomatal flexibility, are needed to understand the costs of Si vs. C investment. One recent study found that elevated CO_2_ decreased leaf Si concentration in native grasses, suggesting that atmospheric C availability may also influence Si vs. C investment (Johnson and Hartley 2017). Interestingly, nonnative grasses did not respond in the same manner, suggesting that native species had greater plasticity in Si response as a result of being adapted to a nutrient‐poor, seasonally arid environment.

Our study suggests that the greatest benefit of silicon accumulation occurs in arid or nutrient‐poor environments. Experimental studies also suggest relationships between water availability and leaf Si. For instance, maize plants fertilized with Si decreased stomatal conductance and transpiration rates (Gao et al. 2004). Likewise, under drought conditions, silicon‐fed *Sorghum* exhibited improved root growth, which allowed plants to extract more water from dry soils (Hattori et al. 2005). Collectively, Si accumulation appears to improve water use efficiency under drought conditions to an extent that outweighs potential costs. At NutNet sites, the relationship between leaf Si and leaf C becomes weak to nonexistent at mesic sites (i.e., Fig. 4 inset) suggesting that the beneficial aspects of grass silicon accumulation dissipate when water is abundant. We hypothesize that under mesic conditions, cell wall rigidity conferred by silicification becomes inconsequential because plants can maintain turgor pressure and water loss via transpiration becomes less costly. Furthermore, our findings suggest that active Si uptake and transport are occurring in addition to passive uptake, because we would expect to observe a positive relationship between leaf Si and water availability if accumulation was only the result of passive uptake via mass flow. Variation in uptake dynamics results in the observed trade‐off between leaf Si and leaf C, which we believe has been driven by differences in functional demands conferred by arid vs. mesic environments.

Although Si accumulation is cited as an inducible herbivore defense, recent evidence suggests that herbivore‐induced Si accumulation is not consistent among species and biomes. In a field experiment excluding mammalian herbivores, four of five grass species showed no significant leaf Si response to exclosures, and site‐level differences in leaf Si were not explained by herbivore density (Soininen et al. 2013). In Serengeti National Park, environmental gradients provide an alternative explanation for regional variation in grass leaf Si (Quigley and Anderson 2014, Quigley et al. 2017) which was initially posited as an adaptive response to variation in grazing pressure (McNaughton and Tarrants 1983, McNaughton et al. 1985). High leaf Si has also been observed for heavily grazed grasslands of the American Midwest, but other environmental conditions were not considered (Brizuela et al. 1986). The efficacy of Si uptake as a short‐term defense against herbivores deserves further skepticism, because successional grazing (in which multiple waves of grazers feed on regrowing, highly palatable grasses) occurs in both the Serengeti and the North American plains (McNaughton 1976, Coppock et al. 1983a, 1983b).

In this study, which included sites ranging from grazing ecosystems dominated by large migratory ungulates (i.e., Serengeti National Park) to old field meadows with low herbivore abundance (i.e., Cowichan), the effects of herbivore exclusion on plant Si were equivocal. Although there was no consistent effect of herbivore exclusion (i.e., fencing) or site herbivore abundance on plant Si concentrations, we acknowledge that many of the NutNet sites studied here are not characterized by high levels of herbivore consumption, and therefore, a strong response to fencing may not be expected. Because uncertainty remains regarding how fences modified grazing rates in this study, we are hesitant to conclude that grasses do not accumulate silica as a defense mechanism in response to herbivory.

Regardless, the strong response of plant foliar Si to climate and nutrients point toward an alternative or parallel functional role of leaf Si, such as in structural support or stress tolerance. Our consideration of grazers was limited to large terrestrial mammals, but leaf silica may deter insect herbivores by accelerating mandibular wear (Jambunathan et al. 2009) or through delayed development (Han et al. 2015) and has also been implicated as a defensive strategy that deters root herbivores (Frew et al. 2017). Additional research is required to understand the relative roles of small vs. large grazers and insect vs. mammalian grazers in Si induction and the global significance of herbivores on leaf silica accumulation. Merging contemporary patterns of plant silicification, phylogenetic analyses, and earth system models, which couple the lithosphere and biosphere, is the next necessary step in understanding ecological and evolutionary drivers of plant Si.
