# Supplementary material for: Soil nutrients and precipitation are major drivers of global patterns of grass leaf silicification
Source: Ecology. 2020 Apr 17;101(6):e03006. doi: 10.1002/ecy.3006 (PMC7317429; doi:10.1002/ecy.3006)
Supplement: Supplementary file 1 [file ECY-101-e03006-s001.pdf]

**Supporting Information.** Quigley, K. M., D. M. Griffith, G. L. Donati, and T. M. Anderson. 2020. Soil nutrients and precipitation are major drivers of global patterns of grass leaf silicification. Ecology.

**Appendix S1**

**Methods S1**

**Analytical methods**

Following sample grinding, all plant samples were oven dried and stored in a dessicator until near infrared spectroscopy (NIRS) analysis. Each sample was then scanned 3 times using a Bruker Multipurpose Analyzer (Bruker Optics Inc., Billerica, MA, USA), recorded as the logarithmic inverse reflectance from 1300 to 2650 nm and utilizing a macrosample rotator (*ca.* 15 mm) when sufficient plant biomass was available. A previous study of plant Si using NIRS suggested that creating densely packed “tablets” of pulverized plant material may improve NIRS prediction, but we compared loose and pelleted plant material and saw no difference in predictive quality (Smis et al. 2014, Quigley et al. 2016).

After NIRS analysis, an autoclave-induced base digestion procedure was used to extract silicon from plant samples. Briefly, plant material was placed in an aqueous solution with $H_2O_2$ and NaOH and digested at high temperature and pressure (126 ˚C, 138 kPa) for 1 hour. Under these conditions, the organic plant tissue matrix is dissolved by NaOH and undergoes oxidation by the peroxide; organic material remains bound to the oxidant, and Si is then released from the matrix and dissolved by NaOH (Elliott & Snyder 1991). Immediately following digestion (< 24hr), 1 ml of 5 mmol $NH_4F$ (Kraska & Breitenbeck 2010) was added to each sample and they were diluted to 50 ml in distilled-deionized water. The fluoride ion added in this step contributes to the complete dissolution of Si compounds, which may not be totally dissolved during the previous strong-base digestion. The resulting solution is then further diluted prior to Si quantification by

inductively couple plasma optical emission spectroscopy (ICP OES). In this case, 100 µl of solution is diluted to 10 ml with 1% v/v HCl. Recovery following digestion was validated by use of a certified reference material (Community Bureau of Reference, BCR, Reference material No. 129, hay powder).

A Prodigy ICP OES system (Teledyne Leeman Labs, Hudson, NH, USA) composed of an automatic sampler, a double-pass spray chamber and a concentric nebulizer was used to quantify Si content of selected calibration samples. The operating conditions were: axial view mode, radio-frequency applied power 1.3 kW, plasma gas flow rate 18 L/min, nebulizer pressure 30 psi, sample flow rate 0.6 mL/min, and atomic emission integration time 15 s, with three replicates per sample. Standard reference solutions were prepared by diluting a silicon stock solution (1000 mg/L, High Purity Standards, Charleston, SC, USA) with distilled-deionized water (18.2 MΩ cm, Milli-Q, Millipore, Bedford, MA, USA) in 1% v/v HCl (Certified ACS Plus, Fisher, Pittsburgh, PA, USA). Atomic emission intensities for Si at 251.611 nm (mean of 3 replicates) were used to build the analytical calibration curve. The limit of detection (LOD) for the ICP OES determination, calculated according to IUPAC's recommendations as 3 times the standard deviation of the blank solution (n = 10) divided by the calibration curve slope, was 0.05% silicon in dry mass. Samples which fell below the LOD were eliminated from our calibration dataset.

**NIRS Model**

We fit our NIRS calibration model using a Partial Least Squares (PLS) regression relating leaf Si to spectral variation, using the "plantspec" R package (Griffith & Anderson 2018). Representative calibration (n = 345) and validation (n = 87) data subsets were selected for wet chemistry (ICP OES) using a modified Kennard-Stone algorithm termed "SPXY" which incorporates variation in both the spectra and the Si values (Snee 1977, Saptoro et al. 2012).

During model development, we considered whether model fit improved when focusing on different continents, plant functional types, or when using a variety of spectral preprocessing steps and specific spectral regions. The model with the lowest Root Mean Squared Error of Prediction (RMSEP = 0.52; 25 latent vectors) was one in which spectra were first preprocessed with Vector Normalization and then restricted to between 7500 to 6100 $cm^{-1}$ and 5450 to 4600 $cm^{-1}$ which corroborate the wavenumbers used by Smis *et al.*, (2014). In addition, the model was greatly improved by the removal of bryophyte and Mt. Caroline (Australia) samples, which each had unique spectral properties. Therefore, when analyzing the final dataset, neither bryophytes nor predicted data from Mt. Caroline were included. The final calibration model was validated on the test set and performed well (validation $R^2$ = 0.83; calibration $R^2$ = 0.86) (Supplementary Figure S1).

**Supplementary Tables**

**Table S1.** Sites included in data analysis. Site locations are displayed in Fig. 1 (main text). The grazing index calculations are described in the methods of the main text. For a detailed description of how environmental variables (MAP, MAT) were extracted, see Anderson *et al.* 2018.

| Site name | Country | Longitude | Latitude | 1st year | Habitat type | MAP (mm * $yr^{-1}$) | MAT (°C) | Grazing index |
|---|---|---|---|---|---|---|---|---|
| Bunchgrass | US | -121.97 | 44.28 | 2007 | montane grassland | 1647 | 5.5 | 8 |
| Burrawan | AU | 151.14 | -27.73 | 2008 | semiarid grassland | 683 | 18.4 | 13 |
| Chichaqua Bottoms | US | -93.21 | 41.79 | 2009 | tallgrass prairie | 855 | 9 | 10 |
| Cowichan | CA | -123.38 | 48.46 | 2007 | old field | 764 | 9.8 | 3 |
| Mt. Gilboa | ZA | 30.29 | -29.28 | 2010 | montane grassland | 926 | 13.1 | 5 |
| Hall's Prairie | US | -86.70 | 36.87 | 2007 | tallgrass prairie | 1282 | 13.6 | 5 |
| Hart Mountain | US | -119.50 | 42.72 | 2007 | shrub steppe | 272 | 7.4 | 11 |
| Lookout | US | -122.41 | 44.21 | 2007 | montane grassland | 1898 | 4.8 | 8 |
| Mar Chiquita | AR | -57.42 | -37.72 | 2012 | Grassland | 838 | 13.9 | 8 |
| Mt. Caroline | AU | 117.61 | -31.78 | 2008 | Savanna | 330 | 17.3 | 9 |
| Sagehen Creek | US | -120.24 | 39.43 | 2007 | montane grassland | 882 | 5.7 | 9 |
| Serengeti | TZ | 34.51 | -2.25 | 2008 | Savanna | 854 | 22.1 | 29 |
| Shortgrass steppe | US | -104.77 | 40.82 | 2007 | shortgrass prairie | 365 | 8.4 | 14 |
| Spindletop | US | -84.50 | 38.14 | 2007 | Pasture | 1140 | 12.5 | 4 |
| Summerveld | ZA | 30.72 | -29.81 | 2009 | mesic grassland | 939 | 18.2 | 5 |
| Ukulinga | ZA | 30.40 | -29.67 | 2009 | mesic grassland | 880 | 18.1 | 0 |
| Val Mustair | CH | 10.37 | 46.63 | 2008 | alpine grassland | 1098 | 0.3 | 9 |

**Table S2.** Complete table of models compared using AIC to predict grass leaf Si, with models arranged according to ΔAIC. $R^2$c represents the coefficient of determination for fixed effects only (conditional), and $R^2$m represents the coeffecient of determination which accounts for a random effect of site (marginal) as described by Nakagawa & Schielzeth (2013). The Akaike weight (wi) indicates the probability that a model from the respective model set is the best one.

| Model components | df | AIC | ΔAIC | $w_i$ | $R^2_M$ | $R^2_C$ |
|---|---|---|---|---|---|---|
| NPK + soil N | 5 | 166.02 | 0.0 | 0.6040 | 0.23 | 0.66 |
| NPK | 4 | 168.64 | 2.5 | 0.1640 | 0.05 | 0.71 |
| NPK + soil C | 5 | 169.60 | 3.6 | 0.1010 | 0.25 | 0.68 |
| NPK * soil N | 6 | 169.60 | 3.7 | 0.1010 | 0.22 | 0.66 |
| FENCE + NPK | 5 | 174.27 | 8.2 | 0.0110 | 0.05 | 0.71 |
| NPK + soil C + soil N + pH | 7 | 174.12 | 8.5 | 0.0100 | 0.23 | 0.69 |
| grazing index + NPK | 5 | 175.32 | 9.3 | 0.0060 | 0.05 | 0.72 |
| FENCE * NPK | 6 | 177.94 | 12.1 | 0.0020 | 0.06 | 0.71 |
| NPK * soil C | 6 | 178.62 | 12.8 | 0.0010 | 0.25 | 0.68 |
| NPK + MAP | 5 | 180.79 | 14.8 | 0.0000 | 0.25 | 0.72 |
| soil N | 4 | 183.11 | 16.9 | 0.0000 | 0.22 | 0.61 |
| pH | 4 | 183.30 | 17.1 | 0.0000 | 0.11 | 0.64 |
| NPK * SAND | 6 | 184.21 | 18.4 | 0.0000 | 0.08 | 0.73 |
| All soil variables | 8 | 184.75 | 19.3 | 0.0000 | 0.23 | 0.69 |
| FENCE + NPK + MAP | 6 | 186.44 | 20.6 | 0.0000 | 0.25 | 0.72 |
| soil C | 4 | 186.93 | 20.8 | 0.0000 | 0.23 | 0.63 |
| MAT | 4 | 192.66 | 26.5 | 0.0000 | 0.12 | 0.67 |
| FENCE | 4 | 193.56 | 27.4 | 0.0000 | 0.00 | 0.07 |
| grazing index | 4 | 194.71 | 28.5 | 0.0000 | 0.00 | 0.67 |
| NPK * MAP | 6 | 196.12 | 30.3 | 0.0000 | 0.25 | 0.73 |
| SAND | 4 | 198.19 | 32.0 | 0.0000 | 0.02 | 0.66 |
| grazing index + MAT | 5 | 198.67 | 32.6 | 0.0000 | 0.15 | 0.68 |
| MAP | 4 | 200.45 | 34.3 | 0.0000 | 0.19 | 0.67 |
| grazing index + SAND | 5 | 203.82 | 37.8 | 0.0000 | 0.03 | 0.66 |
| FENCE + MAP | 5 | 205.98 | 40.0 | 0.0000 | 0.19 | 0.67 |
| MAP + MAT | 5 | 206.60 | 40.6 | 0.0000 | 0.24 | 0.68 |
| grazing index * MAT | 6 | 206.63 | 40.8 | 0.0000 | 0.23 | 0.69 |
| grazing index + MAP | 5 | 207.08 | 41.1 | 0.0000 | 0.19 | 0.68 |
| soil C * SAND | 6 | 208.32 | 42.5 | 0.0000 | 0.19 | 0.65 |
| FENCE * SAND | 6 | 214.84 | 49.0 | 0.0000 | 0.02 | 0.67 |

| | | | | | | |
|---|---|---|---|---|---|---|
| grazing index * SAND | 6 | 215.11 | 49.3 | 0.0000 | 0.11 | 0.62 |
| grazing index * MAP | 6 | 219.99 | 54.1 | 0.0000 | 0.32 | 0.67 |
| FENCE * MAP | 6 | 224.01 | 58.2 | 0.0000 | 0.19 | 0.67 |
| MAP * MAT | 6 | 224.98 | 59.1 | 0.0000 | 0.24 | 0.69 |
| MAP * SAND | 6 | 232.36 | 66.5 | 0.0000 | 0.21 | 0.69 |

**Supplementary Figures**

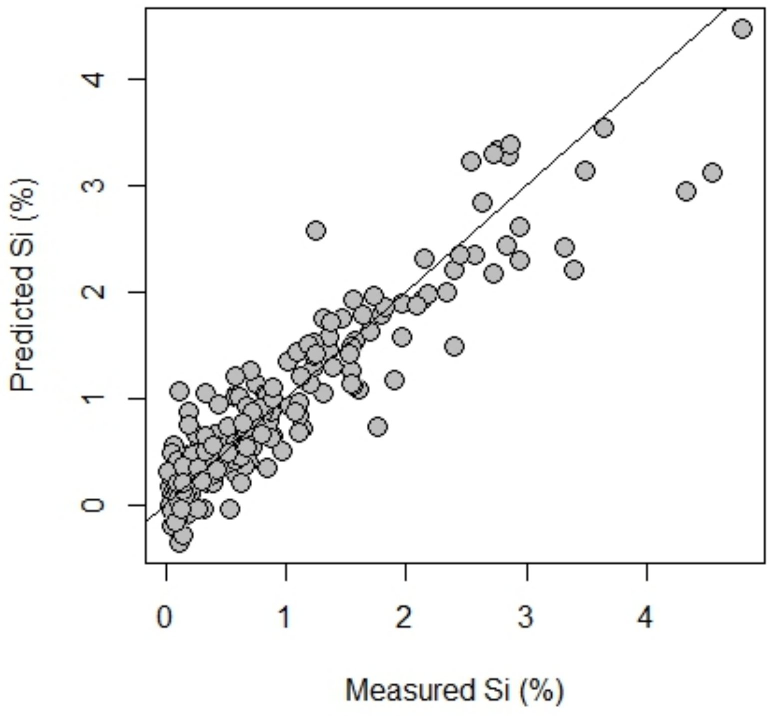


**Figure S1.** Diagnostic plot for NIR Si calibration showing model predictions plotted against measured wet-lab chemistry. This model excluded bryophytes, litter, and samples from a single site (Mt. Caroline).

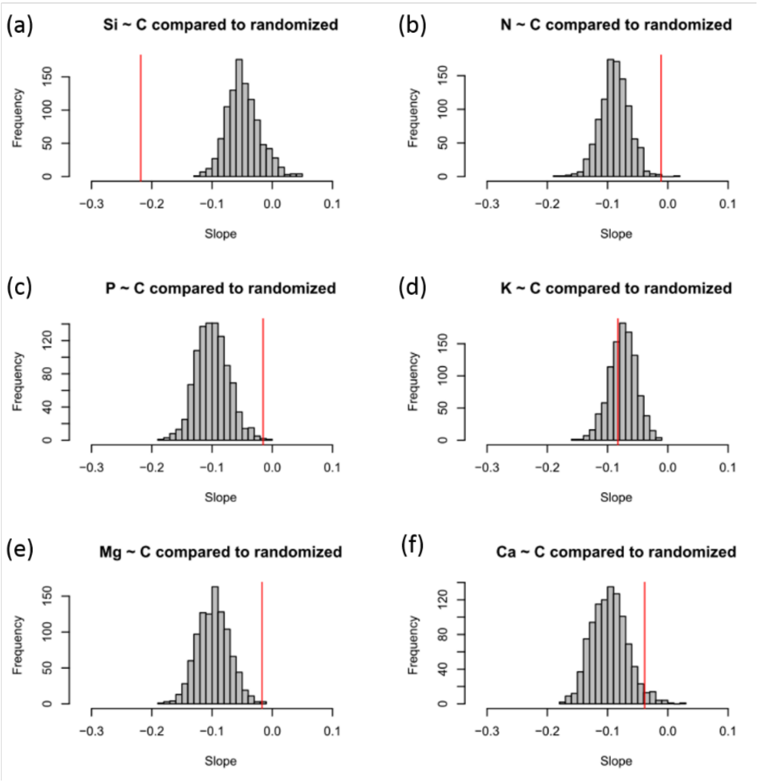


**Figure S2**. Results of the dilution effect model simulations. The red line represents the observed slope for each relationship, and histogram shows the distribution of 999 random slopes (see methods). An observed slope more negative than the expected distribution of slopes indicates that a particular element is being exchanged for carbon.

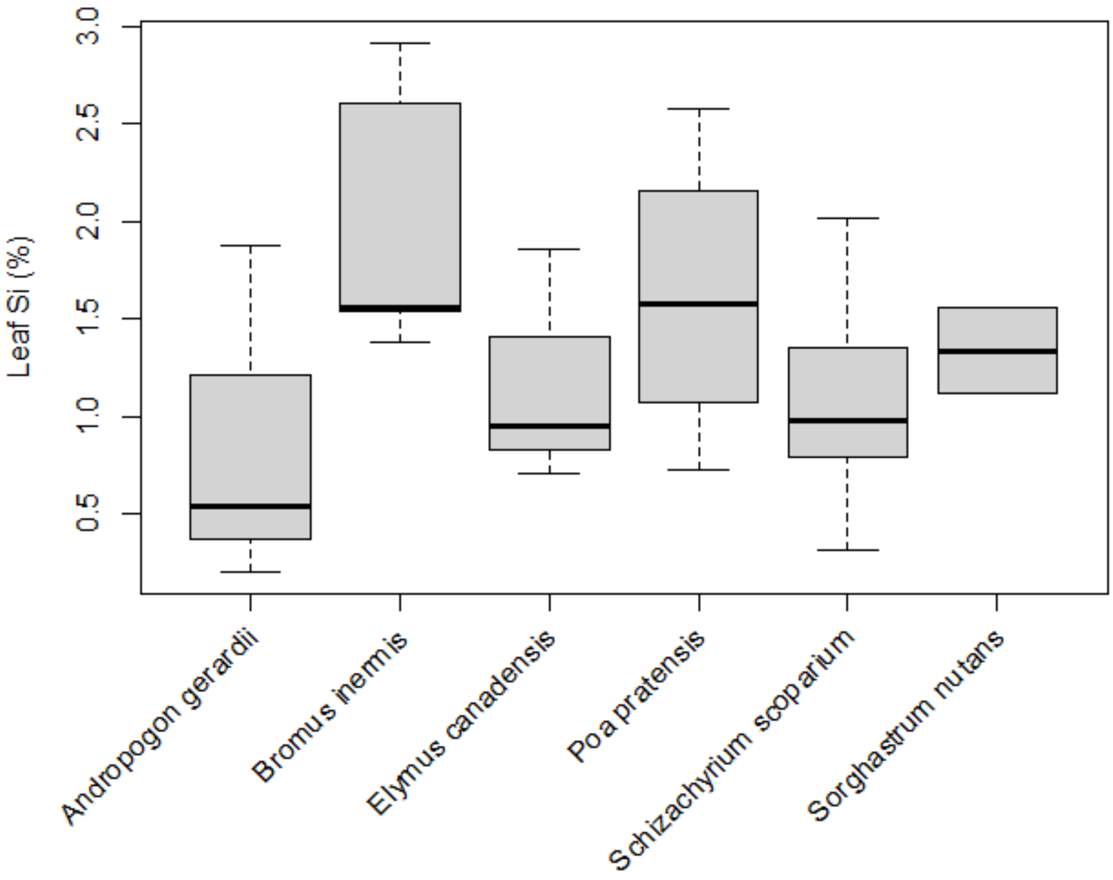


**Figure S3.** Species-specific Si-accumulation of grass species from the site Chichaqua Bottoms. *Andropogon gerardii* is a C4 grass, and the remaining 5 species are all C3.

**Citations**:


Anderson, T.M., D.M. Griffith, J.B. Grace, E.M. Lind, P.B. Adler, L.A. Biederman, D.M. Blumenthal, P. Daleo, J. Firn, N. Hagenah, W.S. Harpole, A.S. MacDougall, R.L. McCulley, S.M. Prober, A.C. Risch, M. Sankaran, M. Schütz, E. Seabloom, L. Sullivan, P. Wragg and E.T. Borer. 2018. Herbivory and eutrophication modulate grassland plant nutrient responses across a global climatic gradient. Ecology 99(4): 822-831.

Griffith, D.M. & Anderson T.M. 2018. The 'plantspec' R package: a tool for spectral analysis of plant stoichiometry. Methods in Ecology and Evolution 10: 673-679.

Nakagawa, S. & Schielzeth, H. 2013. A general and simple method for obtaining $R^2$ from generalized linear mixed-effects models. Methods in Ecology and Evolution 4: 133-142.

Quigley, Kathleen M., Amanda G. Althoff, and George L. Donati. 2016. Inductively Coupled Plasma Optical Emission Spectrometv as a Reference Method for Silicon Estimation by near Infrared Specroscopy and Potential Application to Global-Scale Studies of Plant Chemistry. Microchemical Journal: 129: 231–35.

Saptoro, A., M. O. Tadé, and H. Vuthaluru. 2012. A Modified Kennard-Stone Algorithm for Optimal Division of Data for Developing Artificial Neural Network Models. Chemical Product and Process Modeling 7:1–14.

Smis, Adriaan, Francisco Javier Ancin Murguzur, Eric Struyf, Eeva M Soininen, Juan G Herranz Jusdado, Patrick Meire, and Kari Anne Bråthen. 2014. Determination of Plant Silicon Content with near Infrared Reflectance Spectroscopy. Frontiers in Plant Science 5: 496.

Snee, R. D. 1977. Validation of Regression Models: Methods and Examples. Technometrics 19:415–428.
